# Adsorption of Transition Metals on Black Phosphorene: a First-Principles Study

**DOI:** 10.1186/s11671-018-2696-x

**Published:** 2018-09-12

**Authors:** Yi Luo, Chongdan Ren, Sake Wang, Shaohan Li, Peigen Zhang, Jin Yu, Minglei Sun, Zhengming Sun, Wencheng Tang

**Affiliations:** 1 Jiangsu Key Laboratory of Advanced Metallic Materials, School of Materials Science and Engineering, Southeast University, Nanjing, 211189 Jiangsu People’s Republic of China; 2 Department of Physics, Zunyi Normal College, Zunyi, 563002 Guizhou People’s Republic of China; 3 Department of Fundamental Courses, Jinling Institute of Technology, Nanjing, 211169 Jiangsu People’s Republic of China; 4 School of Mechanical Engineering, Southeast University, Nanjing, 211189 Jiangsu People’s Republic of China; 5 Institute of High Performance Computing, A*STAR, Singapore, 138632 Singapore

**Keywords:** Black phosphorene, Adsorption, Half-metal, Spintronics, CO oxidization, Catalyst

## Abstract

Black phosphorene is a novel two-dimensional material which has unique properties and wide applications. Using first-principles calculations, we investigated the adsorption behavior of 12 different transition metals (TMs; Fe, Co, Ni, Cu, Ru, Rh, Pd, Ag, Os, Ir, Pt, and Au) on phosphorene. Our results showed that all of the adsorption systems have a large binding energy. The Fe-, Co-, and Au-phosphorene systems display magnetic states with magnetic moments of 2, 1, and 0.96 *μ*_B_, respectively, which means that these systems are magnetic semiconductors. Adsorption of oxygen molecules on TM-phosphorene was also investigated. Interestingly, all the O_2_-(TM-phosphorene) systems, except O_2_-(Pd-phosphorene), can elongate the O–O bond, which is critical to their application as catalysts in the oxidation of CO. We also found that the adsorption of O_2_ molecules enables the O_2_-(Fe-, Ni-, Cu-, Ir-, Rh-, Ag-, and Au-phosphorene) systems to become magnetic semiconductors, and it allows O_2_-(Co-phosphorene) to display half-metallic state. Our results are expected to have important implications for phosphorene-based catalysis and spintronics.

## Background

Phosphorene [1–3], a monolayer of phosphorus atoms arranged in a puckered honeycomb structure, has unique properties which include a direct semiconducting nature [4], ultrahigh mobility at room temperature [4–6], superior mechanical flexibility [7], and high thermoelectric performance [8–10]. These properties make phosphorene a very suitable material for a variety of applications such as field-effect transistors [1, 11–16], Li- and Na-ion batteries [17–19], solar cells [20, 21], photocatalysts [22], spintronics [23], and gas sensors [24–26]. However, phosphorene is a nonmagnetic material, and some strategies must be adopted to widen its application.

For two-dimensional (2D) materials, adsorption is usually selected as the approach to induce magnetism for specific applications. Previously, Cao et al. [27] showed that the electronic and magnetic properties of graphene can be effectively modulated by adatoms of Fe, Co, Ni, and Cu. Kaloni et al. [28] demonstrated that magnetic moments can be induced in Ti-, V-, Cr-, Mn-, Fe-, and Co-decorated silicene systems using first-principles calculations. Ersan et al. [29] found that *b*-Arsenene displayed spin-polarized characters after adsorption of H, B, C, P, Ge, As, and Sb atoms. Furthermore, *w*-Arsenene can attain net magnetic moments with the adatoms of H, B, N, P, Cl, Ti, As, and Sb. For black phosphorene, Kulish et al. [30] predicted that Ag-, Au-, Ti-, V-, Cr-, Mn-, Fe-, and Co-phosphorene are rather stable, and a diverse range of magnetic moments can be induced in theoretical calculations. Moreover, the properties of different types of charge carriers can also be tuned by adsorbing different atoms on phosphorene. Ding and Wang [31] used the first-principles calculations to systematically illustrate the structural, electronic, and magnetic properties of atoms adsorbed on phosphorene. They noted that adatoms can introduce magnetism in phosphorene, with P, Co, and Au adatoms inducing stable magnetic properties. Hu and Hong [32] used the first-principles calculations to demonstrate the magnetic properties of metal adatoms on phosphorene; they showed that magnetism can be obtained in phosphorene by adsorbing Cr, Fe, Co, or Au atoms on its surface. Furthermore, they predicted that the Fe-phosphorene adsorption system will be a promising dilute magnetic semiconducting material. Thus, the adsorption of transition metals (TMs) on black phosphorene can be expected to effectively tune the magnetic properties of the material.

Although the above investigations studied the adsorption behavior of transition metals on black phosphorene, some issues remain unresolved. For instance, previous studies mainly focused on the properties of 3d TMs adsorbed on phosphorene. How will 4d and 5d TMs engineer the properties of phosphorene? In addition, noble metals absorbed on phosphorene can also be used as single-atom catalysts. Li et al. [33] suggested that silicene with adsorbed Au can be a high-activity catalyst with low catalytic energy barriers for the oxidization of CO. Can a noble metal absorbed on phosphorene also a good candidate for the oxidization of CO? To answer these questions, we present in this paper the results of a detailed first-principles study on the structural, magnetic, and electronic properties of 12 different types of transition metal atoms adsorbed on black phosphorene. We selected elemental Fe, Co, and Ni, which are ferromagnetic metals in their bulk phase; elemental Cu, which is diamagnetic; and the noble metals Ru, Rh, Pd, Ag, Os, Ir, Pt, and Au, which are very effective for the oxidation of CO [19, 34–45]. We found that phosphorene forms strong bonds with all 12 metals, and all of the TM-phosphorene systems are rather robust. The electronic and magnetic properties of phosphorene can be effectively tuned by the adatoms. Moreover, we also found that most TM-phosphorene adsorption systems are good candidates for the catalyst in the oxidation of CO. The results of this investigation can be used for fundamental studies of phosphorene, and they can also widen its potential application in many important fields.

## Methods/Experimental

Our calculations were based on spin-polarized density functional theory (DFT), and they were performed using the Vienna Ab Initio Simulation Package (VASP) [46, 47] and the generalized gradient approximation (GGA) of the Perdew-Burke-Ernzerhof (PBE) functional [48–50]. The DFT-D3 method of Grimme [51] was used to calculate the van der Waals interaction. An energy cutoff of 400 eV with a plane-wave basis set was employed. In the calculations, the atoms were relaxed until the total energy converged to 1 × 10^−5^ eV and the residual force on each atom was less than 0.01 eV/Å. A large supercell (4 × 3) along the zigzag and armchair directions was used to avoid interactions between neighboring unit cells. The lattice constants were set to *a* = 13.20 Å and *b* = 13.74 Å. We applied a vacuum space of 20 Å in the *z* direction to minimize the interactions between adjacent interlayers. During the optimization, a Monkhorst-Pack [52] *k*-point grid of 3 × 3 × 1 was adopted, and a *k*-point grid of 7 × 7 × 1 was used for the total energy calculations.

## Results and Discussion

**Fig. 1:**
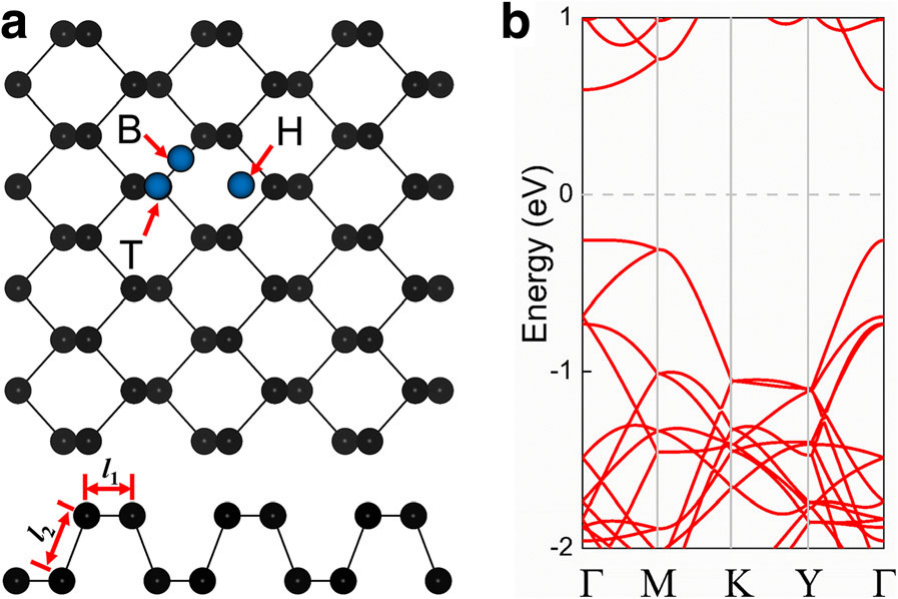
**a** Top and side views of the crystal structure of pristine phosphorene (4 × 3 × 1 supercell). The blue circles represent typical positions of an impurity atom adsorbed at a hollow spot (H), on a bridge (B) between two phosphorus atoms, and on top of a phosphorus atom (T). **b** Electronic band structure and first Brillouin zone of pristine phosphorene; the Fermi level is set to zero

We first explored the structural properties of pristine phosphorene. Figure 1a shows the illustrations of the top and side views of the crystal structure. It can be seen that the phosphorene monolayer consists of two atomic planes, and the unit cell of phosphorene consists of four P atoms. The phosphorene monolayer has a tetragonal lattice with equilibrium lattice constants *a* = 3.30 Å and *b* = 4.58 Å. The length of the P–P bond in the horizontal direction (*l*_1_) is 2.22 Å, while the length in the other direction (*l*_2_) is 2.26 Å. The pristine phosphorene has a direct bandgap of 0.89 eV (Fig. 1b), with both the conduction band minimum (CBM) and the valence band maximum (VBM) located at the Г point. The lattice constant and the bandgap we obtained highly agree with the values obtained in previous research studies [30–32, 53].

A typical adatom is always adsorbed at either one of three positions: above a hollow site (H), on a bridge (B) between two phosphorus atoms, and on top of a phosphorus atom (T). We calculated the adsorption energy of an adatom on phosphorene to examine the stability of the adsorption systems using the relationship:

1 $$ {E}_{\mathrm{ad}}=\left({E}_{\mathrm{TM}}+{E}_{\mathrm{phosphorene}}\right)-{E}_{\mathrm{TM}-\mathrm{phosphorene}} $$

where *E*_TM_ is the energy of an isolated metal atom, *E*_phosphorene_ is the total energy of the pristine phosphorene layer, and *E*_TM-phosphorene_ is the total energy of the adsorption system. Based on this equation, a larger adsorption energy indicates a more stable structure. We found that all the metal atoms studied in our work prefer to stay on the H site of phosphorene. The calculated adsorption energies of metal atoms adsorbed on the H site of phosphorene, shown in Table 1, vary from 2 to 6 eV. The bond length of TM-phosphorene (*d*_TM-P_) was demonstrated to be short, in the range of 2.11–2.43 Å. Bader charge analysis [54–56] shows that 0.16, 0.16, 0.07, 0.17, 0.32, 0.33, and 0.16|e| are transferred from the Ru, Rh, Pd, Os, Ir, Pt, and Au metal atoms, respectively, to phosphorene in the (4d-TM)-phosphorene and (5d-TM)-phosphorene adsorption systems. All these results denote the formation of chemical bonds between the TM adatom and phosphorene. In addition, these results are close to recent studies [30–32].

**Table 1:** Calculated minimum bond length of TM-phosphorene (*d*_TM-P_), adsorption energy (*E*_ad_), total magnetic moment (*M*_total_), and charge transferred from TM adatom to phosphorene for a single TM atom adsorbed at the most stable adsorption site on phosphorene

Adatom	*d*_TM-P_ (Å)	*E*_ad_ (eV)	*M*_total_ (*μ*_B_)	*C* (e)
Fe	2.16	3.254	2.00	− 0.30
Co	2.12	4.158	1.00	− 0.17
Ni	2.11	4.550	0.00	− 0.12
Cu	2.21	2.517	0.00	− 0.29
Ru	2.20	5.32	0.00	+ 0.16
Rh	2.20	5.32	0.00	+ 0.16
Pd	2.26	3.824	0.00	+ 0.07
Ag	2.43	1.465	0.00	− 0.21
Os	2.18	5.547	0.00	+ 0.17
Ir	2.19	5.969	0.00	+ 0.32
Pt	2.22	5.219	0.00	+ 0.33
Au	2.34	1.997	0.96	+ 0.16

**Fig. 2:**
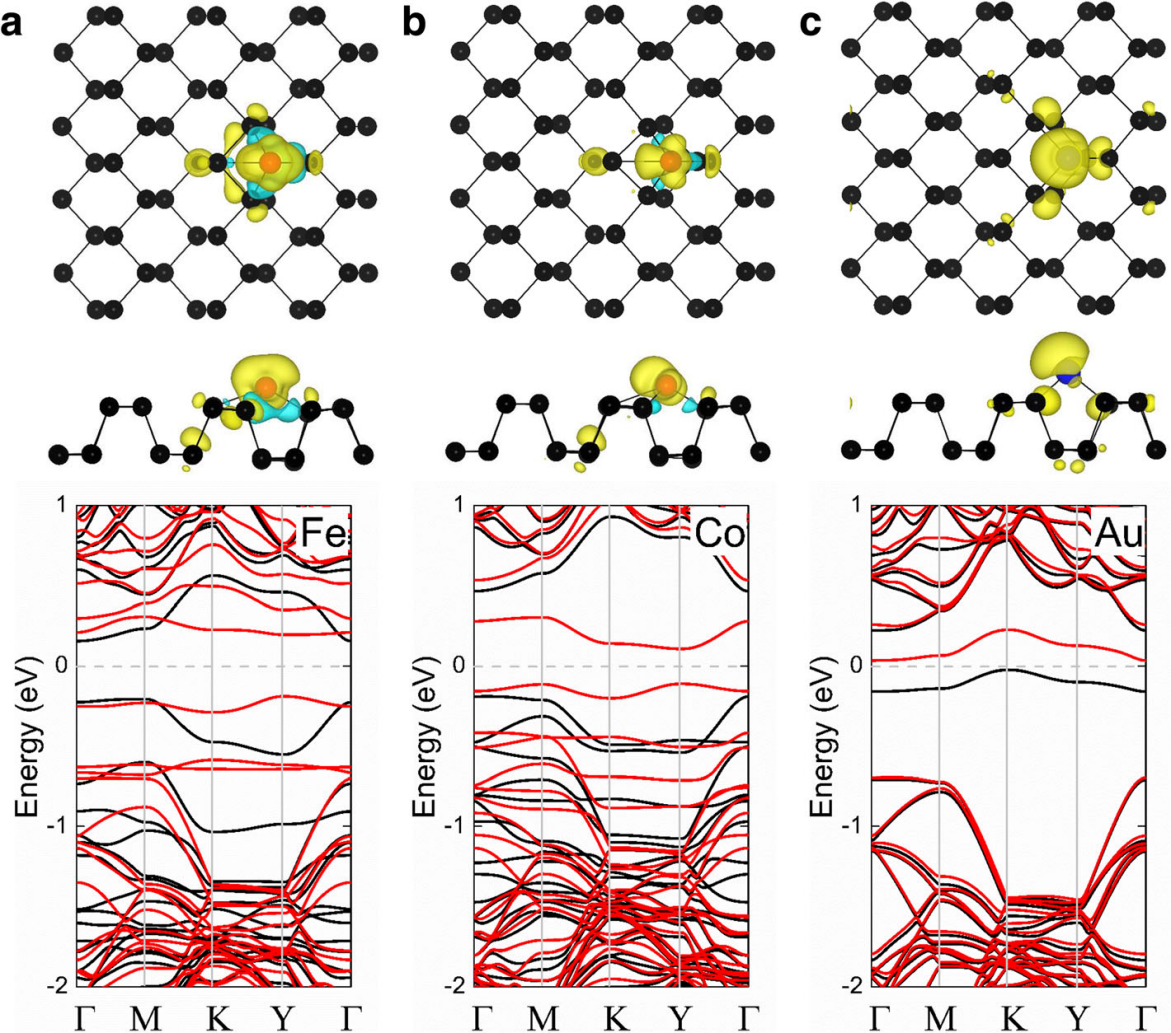
Spin densities of the **a** Fe-phosphorene, **b** Co-phosphorene, and **c** Au-phosphorene systems are shown in the top row; the corresponding band structure of each system is shown at the bottom row. The black and red spheres represent P and TM atoms, respectively. In the top row, a plot of the spin-polarized charge density with a charge density iso-surface value of 0.002 e/Å^3^ is superimposed on the top and side views of the crystal structure of pristine phosphorene for each of the TM-phosphorene systems; the yellow and cyan regions correspond to the up and down spins, respectively. In the plot of band structures (bottom row), the black and the red lines denote spin-up and spin-down channels, respectively; the Fermi level is set to zero, and it is indicated by the gray dashed line

As shown in Table 1, the Ni-, Cu-, Ru-, Rh-, Pd-, Ag-, Os-, Ir-, and Pt-phosphorene systems exhibit nonmagnetic states, while the Fe-, Co-, and Au-phosphorene systems have the magnetic moments of 2, 1, and 0.96 *μ*_B_, respectively. The spin-polarized charge density (*ρ* = *ρ*_spin-up_ − *ρ*_spin-down_) is also shown in Fig. 2 to explore the origin and distribution of magnetism in the magnetic TM-phosphorene adsorption systems. The magnetic moment in each of these cases primarily originates from the adatom, with a small magnetic moment resulting from the nearest neighbors. Furthermore, the calculated band structures of the Fe-, Co-, and Au-phosphorene systems are depicted in Fig. 2. It can be seen that these systems are all magnetic semiconductors with bandgaps of 0.38, 0.22, and 0.06 eV, respectively, which are useful for spintronic applications.

**Fig. 3:**
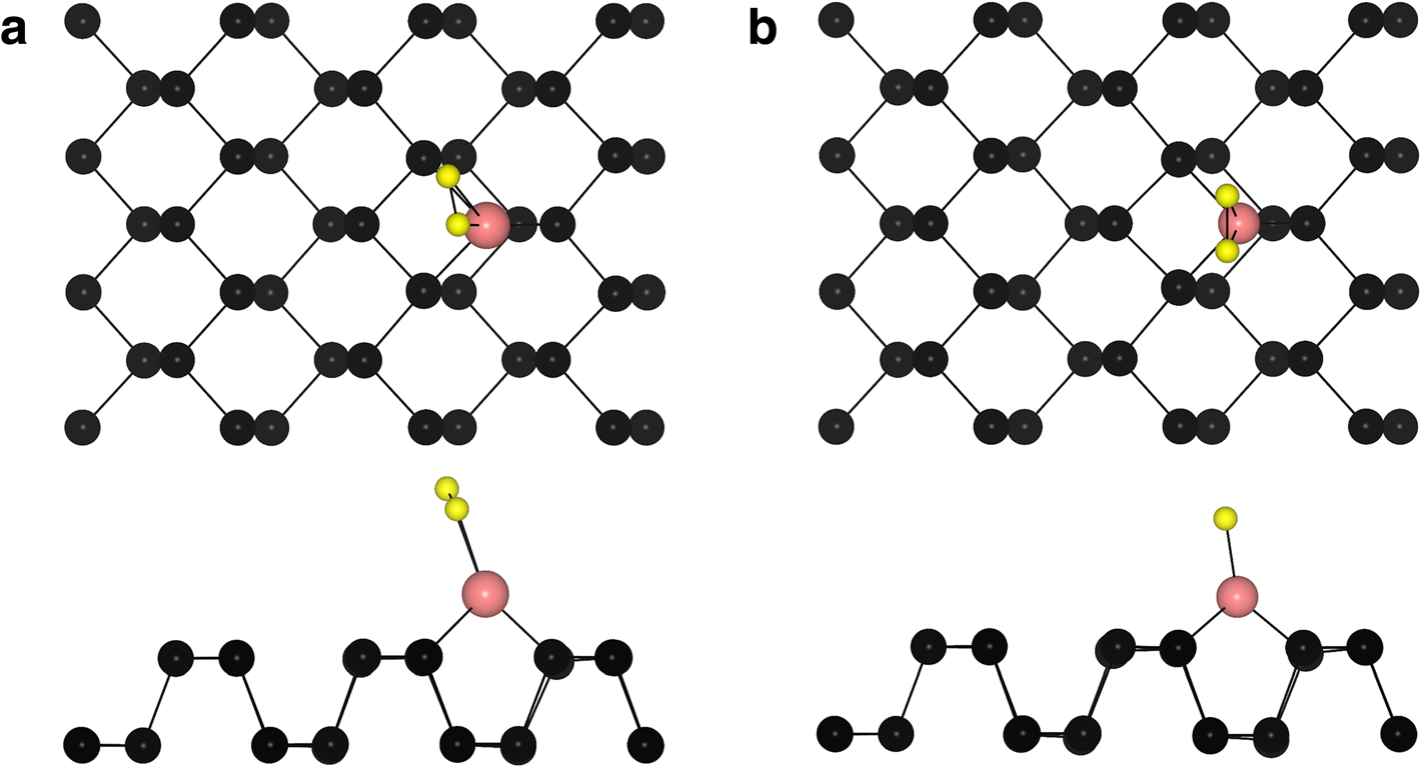
Top and side views of typical adsorption sites of an O_2_ molecule on TM-phosphorene. The black, pink, and yellow spheres represent P, TM, and O atoms, respectively

Next, we studied the adsorption behavior of O_2_ on top of the TM atom in the TM-phosphorene systems. Two typical energy-lowest configurations for the adsorption of O_2_ on TM-phosphorene systems (O_2_-(TM-phosphorene)) are shown in Fig. 3. For O_2_-(Fe-phosphorene), O_2_-(Co-phosphorene), O_2_-(Cu-phosphorene), O_2_-(Pd-phosphorene), and O_2_-(Pt-phosphorene) systems, the O_2_ molecule is parallel to the zigzag direction of phosphorene (Fig. 3a), with an O–P bond length of 1.84 Å, 1.86 Å, 2.04 Å, 2.18 Å, and 2.05 Å, respectively. For the O_2_-(Ni-phosphorene), O_2_-(Ru-phosphorene), O_2_-(Rh-phosphorene), O_2_-(Ag-phosphorene), O_2_-(Os-phosphorene), O_2_-(Ir-phosphorene), and O_2_-(Au-phosphorene) systems, the molecule is along the zigzag direction of phosphorene (Fig. 3b), at a certain angle from the surface. Meanwhile, the two neighboring O atoms around the TM adatom are not equivalent. The results are displayed in Table 2. The adsorption energy (*E*_ad_) of O_2_ on an O_2_-(TM-phosphorene) system was calculated as:

2 $$ {E}_{\mathrm{ad}}={E}_{\mathrm{TM}-\mathrm{phosphorene}}+{E}_{{\mathrm{O}}^2}-{E}_{{\mathrm{O}}^2-\mathrm{TM}-\mathrm{phosphorene}} $$

where ${E}_{{\mathrm{O}}^2-\mathrm{TM}-\mathrm{phosphorene}}$, *E*_TM-phosphorene_, and ${E}_{{\mathrm{O}}^2}$ are the total energies of the O_2_-(TM-phosphorene) system, the TM-phosphorene system, and the O_2_ molecule, respectively. As shown in Table 2, the adsorption energies are 2.659, 1.850, 0.970, 0.906, 2.402, 1.548, 0.001, 0.786, 3.109, 1.980, 0.416, and 1.029 eV for the O_2_-(Fe-phosphorene), O_2_-(Co-phosphorene), O_2_-(Ni-phosphorene), O_2_-(Cu-phosphorene), O_2_-(Ru-phosphorene), O_2_-(Rh-phosphorene), O_2_-(Pd-phosphorene), O_2_-(Ag-phosphorene), O_2_-(Os-phosphorene), O_2_-(Ir-phosphorene), O_2_-(Pt-phosphorene), and O_2_-(Au-phosphorene) systems, respectively. In all cases, the large adsorption energies except for that of the O_2_-(Pd-phosphorene) system indicate that O_2_ is chemisorbed.

**Table 2:** Parameters of O_2_-(TM-phosphorene) adsorption systems: adsorption energy, charge transferred (*C*) from TM-phosphorene to the O_2_ molecule, and calculated bond lengths of O–O and O–TM

Dopant	*E*_ad_ (eV)	*C* (e)	Bond length (Å)		
			(*d*_O-O_)	(*d*_O1-TM_)	(*d*_O2-TM_)
Fe	2.659	− 0.68	1.38	1.84	1.84
Co	1.850	− 0.50	1.36	1.86	1.86
Ni	0.970	− 0.42	1.32	2.14	1.90
Cu	0.906	− 0.52	1.35	2.04	2.04
Ru	2.402	− 0.46	1.40	1.91	2.08
Rh	1.548	− 0.24	1.34	2.07	2.03
Pd	0.001	− 0.24	1.32	2.18	2.18
Ag	0.786	− 0.37	1.30	2.19	2.98
Os	3.109	− 0.53	1.46	2.04	1.92
Ir	1.980	− 0.25	1.39	2.00	2.06
Pt	0.416	− 0.19	1.40	2.05	2.05
Au	1.029	− 0.09	1.32	2.12	2.93

It is fairly recognized that the elongation of the O–O bond is crucial for both Langmuir-Hinshelwood and Eley-Rideal mechanisms of a catalyst in the oxidation of CO [57]. Generally speaking, the longer the O–O bond length, the easier the catalyst reaction. The O–O and TM–O bond lengths in each system are also shown in Table 2. Obviously, the O–O bond increases from 1.23 Å for the pristine O_2_ molecule to 1.38, 1.36, 1.32, 1.35, 1.40, 1.34, 1.32, 1.30, 1.46, 1.39, 1.40, and 1.32 Å, respectively, for the adsorbed molecule, possibly because O_2_ is an electron acceptor. Furthermore, the bond length of TM–O in most O_2_-(TM-phosphorene) systems is short owing to the interaction between O_2_ and the TM atoms. This bond length varies from 1.84 to 2.19 Å and results in the formation of chemical bonds. In particular, the O–O bond is elongated to 1.40 Å, the highest value among the systems, in the adsorbed O_2_ molecule on the Pt-phosphorene system. Thus, the Pt-phosphorene system is quite suitable as a catalyst for the oxidation of CO because it probably has the high catalytic ability.

**Fig. 4:**
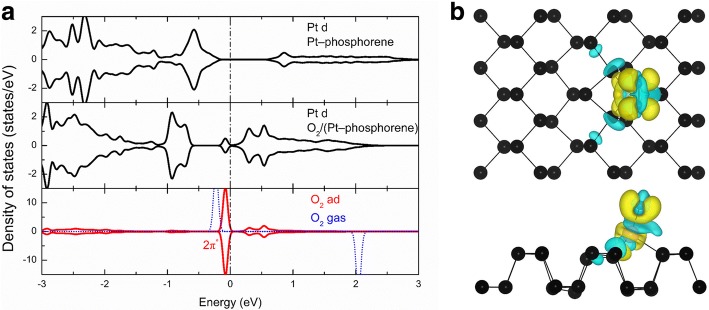
**a** Local density of states (LDOS) of Pt and O_2_ molecules in Pt-phosphorene and O_2_-Pt-phosphorene systems and gas phase O_2_, respectively. **b** Charge density difference in the O_2_-(Pt-phosphorene) system; the yellow region (i.e., + 0.002 e/Å^3^) and the cyan region (i.e., − 0.002 e/Å^3^) correspond to the increase and the loss, respectively, of the electron density

In order to obtain more insight into the underlying mechanism of the high activity of these systems, we selected O_2_-(Pt-phosphorene) as an example and investigated its local density of states (LDOS). Figure 4a shows the LDOS projected onto d orbitals of Pt in the Pt-phosphorene system, d orbitals of Pt in the O_2_-(Pt-phosphorene) system, the O–O bond in the O_2_-(Pt-phosphorene) system, and the gas phase O_2_. In the upper panel of Fig. 4a, one peak can be seen at *E*_F_ − 0.6 eV, which originates from the partially occupied d orbital of Pt in the Pt-phosphorene system. These states should be responsible for the high activity of the Pt-phosphorene system. After the adsorption of an O_2_ molecule, the LDOS projected onto d orbitals of Pt below the Fermi level is downshifted after the adsorption of the O_2_ molecule owing to the charge transfer, and the states above the Fermi level is also substantially increased. Meanwhile, the LDOS projected onto the adsorbed O_2_ molecule indicates that the O_2_ 2π^*^ orbitals (lowest unoccupied molecular orbital, LUMO) are becoming partially occupied, which has downshifted from its gas value of *E*_F_ + 2 eV to *E*_F_ − 0.1 eV. For clarification, the charge density difference of the O_2_-(Pt-phosphorene) system is also presented.

The charge density difference is defined as follows:

3 $$ {\varDelta}_{\rho }={\rho}_T-{\rho}_{\mathrm{molecule}}-{\rho}_{\mathrm{absorbed}} $$

where *ρ*_T_, *ρ*_molecule_, and *ρ*_absorbed_ are the total charges on the O_2_-(Pt-phosphorene) system, O_2_ molecule, and the Pt-phosphorene system, respectively. As shown in Fig. 4b, the large yellow region localized on the O_2_ molecule indicates that there is a significant electron transfer from Pt-phosphorene to O_2_, which also indicates the strong orbital hybridization between O_2_ and the Pt-phosphorene system. According to the Bader charge analysis [54–56], 0.19|e| is transferred from the Pt-phosphorene system to the O_2_ molecule. Therefore, the large charge transfer fills the antibonding states of the O_2_ molecule and significantly weakens the O–O bond. Similarly, the underlying mechanism of the high activity of other systems can also be understood by the charge transfer between the O_2_ molecule and the TM-phosphorene system. Indeed, Bader charge analysis [54–56] showed that charges of − 0.68, − 0.50, − 0.42, − 0.52, − 0.46, − 0.24, − 0.24, − 0.37, − 0.53, − 0.25, − 0.19, and − 0.09|e| are transferred from TM-phosphorene to the oxygen molecule in the O_2_-(Fe-phosphorene), O_2_-(Co-phosphorene), O_2_-(Ni-phosphorene), O_2_-(Cu-phosphorene), O_2_-(Ru-phosphorene), O_2_-(Rh-phosphorene), O_2_-(Pd-phosphorene), O_2_-(Ag-phosphorene), O_2_-(Os-phosphorene), O_2_-(Ir-phosphorene), O_2_-(Pt-phosphorene), and O_2_-(Au-phosphorene) systems, respectively.

**Fig. 5:**
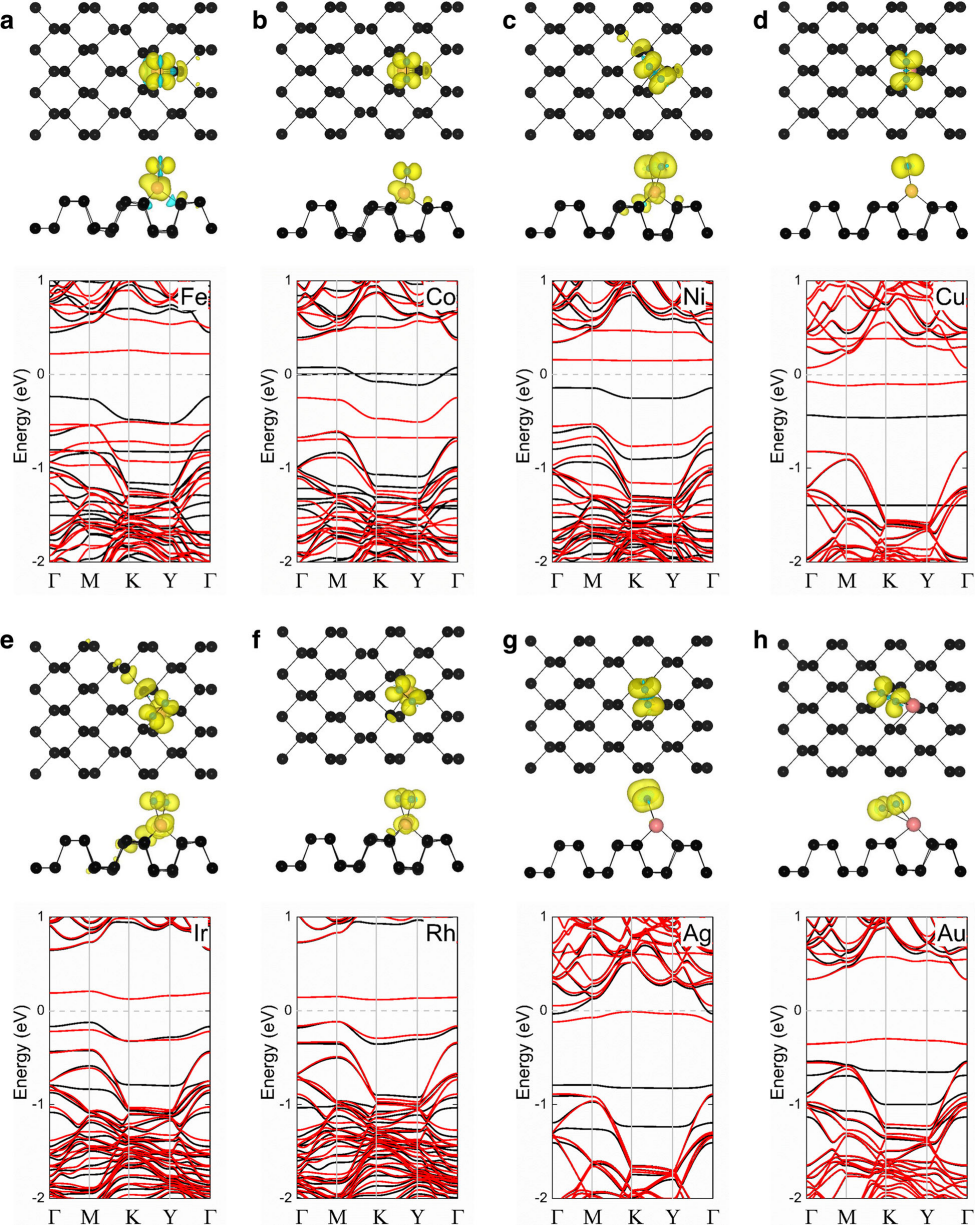
Spin densities of the **a** O_2_-(Fe-phosphorene), **b** O_2_-(Co-phosphorene), **c** O_2_-(Ni-phosphorene), **d** O_2_-(Cu-phosphorene), **e** O_2_-(Ir-phosphorene), **f** O_2_-(Rh-phosphorene), **g** O_2_-(Ag-phosphorene), and **h** O_2_-(Au-phosphorene) systems are shown in the top row; the corresponding band structure of each system is shown in the bottom row. In the top row, a plot of the spin-polarized charge density with a charge density iso-surface value of 0.002 e/Å^3^ is superimposed on the top and side views of the crystal structure of pristine phosphorene; the yellow and cyan regions correspond to up and down spins, respectively. In the plots of band structures, the black and the red lines denote spin-up and spin-down channels, respectively; the Fermi level is set to zero, and it is indicated by the gray dashed line

Finally, we studied the magnetic properties of O_2_-(TM-phosphorene) systems. The magnetic moments of the O_2_-(TM-phosphorene) systems are shown in Table 3. The O_2_-(Ni-phosphorene), O_2_-(Cu-phosphorene), O_2_-(Rh-phosphorene), O_2_-(Ag-phosphorene), and O_2_-(Ir-phosphorene) systems have magnetic moments of 2.00, 1.00, 1.00, 1.14, and 1.00 *μ*_B_, respectively, which all result from the adsorption of a paramagnetic O_2_ molecule. The spin-polarized charge density of these O_2_-(TM-phosphorene) systems is displayed in Fig. 5. For the O_2_-(Fe-phosphorene) and O_2_-(Co-phosphorene) systems, the magnetic moment is believed to mainly arise from the transition metal atom and the O_2_ molecule. On the contrary, for the O_2_-(Ni-phosphorene), O_2_-(Cu-phosphorene), O_2_-(Rh-phosphorene), O_2_-(Ag-phosphorene), O_2_-(Ir-phosphorene), and O_2_-(Au-phosphorene) systems, the magnetic moment mainly comes from the O_2_ molecule. These hypotheses are consistent with the results displayed in Table 3. To better comprehend how the adsorption of a gas molecule affects the electronic structure of the O_2_-(TM-phosphorene) system, the electronic band structures of each system was calculated, and the results are shown in Fig. 5. First, we discovered that a flat band occurs around the Fermi level (*E*_F_) after the adsorption of O_2_ molecule in all systems, which primarily from the O_2_ molecule. For the O_2_-(Fe-phosphorene), O_2_-(Co-phosphorene), O_2_-(Ni-phosphorene), O_2_-(Cu-phosphorene), O_2_-(Rh-phosphorene), O_2_-(Ir-phosphorene), O_2_-(Ag-phosphorene), and O_2_-(Au-phosphorene) systems, the channels for spin-up and spin-down split reveal the magnetic characteristics. The O_2_-(Fe-phosphorene), O_2_- (Ni-phosphorene), O_2_-(Cu-phosphorene), O_2_-(Ir-phosphorene), O_2_-(Rh-phosphorene), O_2_-(Ag-phosphorene), and O_2_-(Au-phosphorene) exhibit magnetic semiconducting behavior, with a considerable bandgap except for the O_2_-(Co-phosphorene) system, which was revealed to be half-metallic. These results suggest that the systems have the potential for application in phosphorene-based spintronics.

**Table 3:** Calculated total magnetic moment (*M*_total_) of O_2_-(TM-phosphorene) systems. The magnetic moments of impurity atoms (*M*_TM_) and an oxygen molecule (${M}_{{\mathrm{O}}^2}$) are also shown for comparison

Dopant	*M*_total_ (*μ*_B_)	*M*_TM_	${M}_{{\mathrm{O}}^2}$
Fe	2.00	1.43	0.51
Co	1.00	0.47	0.44
Ni	2.00	0.49	1.33
Cu	1.00	0.02	1.09
Rh	1.00	0.18	0.76
Ag	1.14	− 0.01	1.33
Ir	1.00	0.27	0.49
Au	1.00	0.00	1.02

## Conclusions

We investigated the structural, electronic, and magnetic properties of different TM-phosphorene systems. All the adatoms were found to prefer to occupy the hollow site on phosphorene. The considerable adsorption energy reveals that all of the TM-phosphorene adsorption systems are rather robust, indicating that phosphorene forms strong bonds with all 12 types of TM adatoms. Furthermore, we found that doping with Fe, Co, and Au can result in magnetic semiconducting properties in monolayered phosphorene, with total magnetic moments of 2, 1, and 0.96 *μ*_B_, respectively.

In addition, we also examined the properties of an O_2_ molecule adsorbed on the TM-phosphorene system. It was very encouraging to find that all of the O_2_-(TM-phosphorene) systems, except for O_2_-(Pd-phosphorene), display good catalytic activity for the oxidation of CO owing to the elongation of the O–O bond. The O_2_-(Fe-phosphorene), O_2_-(Ni-phosphorene), O_2_-(Cu-phosphorene), O_2_-(Rh-phosphorene), O_2_-(Ag-phosphorene), O_2_-(Ir-phosphorene), and O_2_-(Au-phosphorene) systems display spin-polarized semiconducting properties with magnetic moments of 2.00, 2.00, 1.00, 1.00, 1.14, 1.00, and 1.00 *μ*_B_. The O_2_-(Co-phosphorene) displays magnetic half-metallic characteristics, with a magnetic moment of 2.00 *μ*_B_. Therefore, our results may open new possibilities for applying phosphorene in the fields of catalysis and spintronics.

## Abbreviations

2D: Two-dimensional
B: Bridge
GGA: Generalized gradient approximation
H: Hollow site
LDOS: Local density of states
PBE: Perdew-Burke-Ernzerh
T: Top of a phosphorus atom
TM: Transition metal
